# Response to Dobutamine in Septic Shock With Myocardial Dysfunction: A Single-Center Retrospective Study

**DOI:** 10.1097/CCE.0000000000001487

**Published:** 2026-09-25

**Authors:** Marwa Tarbaghia, Abdelrahman Nanah, Ryota Sato, Filippo Sanfilippo, Siddharth Dugar

**Affiliations:** 1 Department of Internal Medicine, Integrated Hospital Institute, Cleveland Clinic, Cleveland, OH.; 2 Department of Pulmonary and Critical Care Medicine, Integrated Hospital Institute, Cleveland Clinic, Cleveland, OH.; 3 Division of Critical Care Medicine, Department of Medicine, The Queen’s Medical Center, Honolulu, HI.; 4 Department of Anaesthesia and Intensive Care, A.O.U. Policlinico-San Marco, Catania, Italy.; 5 Department of Surgery and Medical-Surgical Specialties, University of Catania, Catania, Italy.; 6 Department of Medicine, Cleveland Clinic Lerner College of Medicine, Case Western Reserve University, Cleveland, OH.

**Keywords:** critical illness, dobutamine, echocardiography, hemodynamics, septic, shock

## Abstract

**IMPORTANCE:**

Dobutamine is recommended for septic shock with myocardial dysfunction and persistent hypoperfusion despite preload and mean arterial pressure optimization; however, supporting evidence is limited, and predictors of response remain unclear.

**OBJECTIVES:**

To determine whether early hemodynamic responsiveness to dobutamine is associated with clinical outcomes and whether baseline echocardiographic parameters identify responders.

**DESIGN, SETTING, AND PARTICIPANTS:**

Single-center retrospective cohort study at a quaternary academic medical center. Adults with septic shock who received concomitant dobutamine with vasopressor therapy and underwent transthoracic echocardiography within 12 hours before dobutamine initiation.

**MAIN OUTCOMES AND MEASURES:**

Hemodynamic responsiveness was defined as the combination of an increase in central venous oxygen saturation (ScvO_2_) of at least five percentage points and a reduction in norepinephrine-equivalent (NEE) dose measured at 2 hours after dobutamine initiation. The primary outcome was 28-day ICU mortality. Secondary outcomes included ICU-free days and renal replacement therapy (RRT) requirement.

**RESULTS:**

Of 104 patients, 37 (36%) met the composite definition of hemodynamic response. Baseline demographics, illness severity, lactate, and baseline vasopressor dose were similar between groups. Beyond the 2-hour window used to define response, separation persisted at 6 hours: responders showed a greater reduction in NEE dose (ΔNEE, −0.13 vs. 0.04 µg/kg/min; *p* < 0.001) and a higher MAP/NEE ratio (380 vs. 166; *p* = 0.003), and a greater increase in ScvO_2_ (+12% vs. +6%; *p* = 0.014). 28-day ICU mortality was 41% in responders and 58% in nonresponders (*p* = 0.13); after multivariable adjustment, hemodynamic response was associated with lower 28-day ICU mortality (odds ratio, 0.18; 95% CI, 0.04–0.68; *p* = 0.018). Responders had more ICU-free days (17 [0–22] vs. 0 [0–15]; *p* = 0.017); RRT requirement did not differ (43% vs. 37%; *p* = 0.7). No baseline echocardiographic parameter was associated with hemodynamic responsiveness.

**CONCLUSIONS AND RELEVANCE:**

In septic shock with myocardial dysfunction, early hemodynamic responsiveness to dobutamine identified a subgroup with a more favorable clinical trajectory. Resting echocardiographic parameters did not identify responders. These findings are hypothesis-generating and require prospective confirmation.

**KEY POINTS**

**Question:** Does early hemodynamic responsiveness to dobutamine predict outcomes in septic shock with myocardial dysfunction, and do baseline echocardiographic parameters identify hemodynamic responders?

**Findings:** In this single-center retrospective cohort study of 104 adults with septic shock receiving concomitant dobutamine with vasopressors, 36% were identified as hemodynamic responders. Responders had more ICU-free days and lower odds of adjusted 28-day ICU mortality. No baseline echocardiographic parameter discriminated responders from nonresponders.

**Meaning:** Early hemodynamic response to dobutamine identifies a subgroup with a more favorable shock trajectory, and resting echocardiographic parameters do not identify likely responders.

Sepsis remains a major cause of critical illness and mortality worldwide, defined as a life-threatening organ dysfunction caused by a dysregulated host response to infection (1). Although sepsis most commonly manifests as loss of vasomotor tone with resultant hypotension, a proportion of patients develop concomitant intrinsic myocardial dysfunction, commonly referred to as sepsis-induced myocardial dysfunction, a clinical construct that remains heterogeneous and lacks a standardized, consensus definition (2, 3).

Left ventricular (LV) systolic dysfunction represents the most described phenotype of septic cardiomyopathy. In a systematic review, new-onset LV systolic dysfunction occurred in 20% of septic patients and was associated with significantly higher short-term mortality (3). In another study, LV systolic dysfunction was identified in approximately 18% of mechanically ventilated patients with septic shock at ICU admission, with a proportion developing dysfunction within the first 72 hours after vasopressor initiation (4). Of note, septic patients admitted to ICU may have preexisting cardiac impairment, but the characterization of their baseline cardiac function is rarely available. Preexisting heart failure (HF) serves as a major negative prognostic factor in sepsis. Alon et al (5) demonstrated this disparity, noting a 51% mortality rate in septic patients with preexisting HF compared with 41% in those without (*p* = 0.015). The presence of cardiac dysfunction in sepsis is also associated with increased vasopressor requirements, multiorgan dysfunction, in addition to higher mortality (6–8).

In patients with impaired LV systolic function, targeted management of myocardial dysfunction with inotropic support may theoretically enhance tissue perfusion, reduce organ dysfunction and, consequently, have positive effects on morbidity and mortality. The current Surviving Sepsis Campaign guidelines suggest dobutamine for septic patients with cardiac dysfunction and persistent hypoperfusion despite adequate preload and stabilized mean arterial pressure (MAP), but this suggestion is based on low-quality evidence (9).

In normal subjects, dobutamine increases cardiac output (CO) by increasing myocardial contractility (10). However, in septic shock, vasodilation and hypotension are frequently encountered, and an increase in cardiac contractility is not consistent among patients (11). In addition, adrenergic responsiveness to dobutamine may be blunted in septic patients (12). Previous studies have demonstrated that a substantial proportion of patients with septic shock and impaired LV systolic function are unable to tolerate dobutamine, often due to its predominant vasodilatory effects outweighing the augmentation of myocardial contractility (13). Current literature has yet to establish a clear demographic, hemodynamic, or echocardiographic phenotype that distinguishes patients who will experience improvements in clinical outcomes with dobutamine.

Accordingly, we sought to determine whether clinical outcomes differ between dobutamine responders and nonresponders, and if any baseline echocardiographic and physiologic parameters might predict hemodynamic response following dobutamine initiation.

## MATERIALS AND METHODS

### Ethical Approval

This study was approved by the Cleveland Clinic Institutional Review Board (IRB 15-1233: Hemodynamics in sepsis in critically ill patients, approval date September 29, 2025) with a waiver of informed consent owing to the retrospective design and use of de-identified data. The study was conducted in accordance with the ethical standards of the Declaration of Helsinki.

This was a single-center, retrospective cohort study conducted in the medical ICU of a quaternary academic medical center, from 2011 through 2020. Adult patients (≥ 18 yr) admitted to the ICU with septic shock, as defined by the Sepsis-3.0 criteria (suspected or confirmed infection, vasopressor requirement to maintain a MAP greater than or equal to 65 mm Hg, and serum lactate level greater than 2 mmol/L despite adequate fluid resuscitation), were screened for inclusion (1).

To minimize confounding of echocardiographic parameters, we only included patients with an echocardiogram performed during catecholamine administration and within 12 hours before dobutamine initiation. Dobutamine was initiated at the treating physician’s discretion based on clinical assessment of hemodynamic compromise in the setting of suspected myocardial dysfunction. No predefined echocardiographic threshold was required for inclusion. Patients were excluded if they were noted to have severe valvular disease, a history of heart or lung transplantation, or moderate or large pericardial effusion to ensure accuracy of systolic and diastolic assessment. Patients were also excluded if hourly vasopressor and inotrope data were unavailable, if dobutamine was initiated in the absence of concurrent vasopressor therapy, or if it was discontinued within 6 hours of initiation. The 6-hour duration was defined a priori to ensure that each patient received an adequate trial of dobutamine. Baseline characteristics and outcomes of dobutamine recipients excluded from the analytic cohort were compared with those of included patients (**eTable S1**, https://links.lww.com/CCX/B687). Patients in whom ScvO_2_ at 2 hours was unavailable (*n* = 10) could not be classified and were also excluded. Demographics, comorbidities, illness severity scores, as well as hemodynamic, echocardiographic, and laboratory values at ICU admission were obtained from the electronic medical record.

The echocardiogram was performed at the treating physician’s discretion for clinical indication. All echocardiograms were performed by registered cardiac sonographers using commercially available ultrasound systems. All echocardiographic measurements were obtained offline by board-certified cardiologists using Syngo Dynamics (Siemens Healthcare, Malvern, PA). All parameters were measured according to guidelines from the ASE (14, 15). Stroke volume (SV) was estimated by measuring the left ventricular outflow tract (LVOT) velocity–time integral (VTI) with pulsed-wave Doppler and the LVOT cross-sectional area derived from the LVOT diameter. Arterial elastance (Ea) was calculated as follows: 0.9 × [systolic blood pressure (SBP)]/SV. The LV end-systolic elastance (Ees) was calculated by a simplified approach as follows: 0.9 × SBP/LV end-systolic volume. We calculated ventriculo-arterial coupling as Ea/Ees (16). Systemic vascular resistance (SVR) was calculated as follows: [MAP – central venous pressure (CVP)] × 80/CO, where CVP was estimated based on ASE recommendations.

Vasopressor requirements were quantified as norepinephrine-equivalent (NEE) doses at 0, 2-, and 6-hour time points following dobutamine initiation. NEE was calculated as follows: NEE (µg/kg/min) = norepinephrine (µg/kg/min) + epinephrine (µg/kg/min) + (dopamine [µg/kg/min] ÷ 100) + (phenylephrine [µg/kg/min] ÷ 10) + (vasopressin [U/min] × 2.5) (17). Patients were grouped into dobutamine responders, defined by the presence of both an increase in ScvO_2_ of at least five percentage points and a decrease in NEE dose at 2 hours after dobutamine initiation, and nonresponders as those not meeting both criteria. A composite definition was adopted so that a reduction in vasopressor requirement is accompanied by objective evidence of improved oxygen delivery rather than being interpreted in isolation. The threshold of five percentage points was selected as studies of dobutamine in septic shock report an increase in central and mixed venous oxygen saturation of approximately this magnitude (11, 18, 19). The 2-hour timepoint was selected because the hemodynamic effect of a dobutamine infusion is established within this interval.

The primary outcome was 28-day ICU mortality from initiation of dobutamine. Other outcomes explored included ICU-free days at day 28, acute kidney injury requiring renal replacement therapy (RRT), and overall hospital mortality. In addition, we examined associations between baseline echocardiographic and clinical variables and hemodynamic responsiveness to dobutamine.

### Statistical Analysis

Patients’ characteristics are summarized as median and interquartile range (IQR, 25–75%) for continuous variables. Frequencies and percentages are shown for categorical variables. The Mann-Whitney *U* test was used for continuous variables and the chi-square or Fisher exact test for categorical variables. A multivariable logistic regression model was constructed to assess the independent association between hemodynamic response and 28-day ICU mortality. Covariates were prespecified based on clinical relevance and previous evidence establishing them as predictors of adverse outcomes in septic shock. These included the Acute Physiology and Chronic Health Evaluation (APACHE) III score, mechanical ventilation, acute kidney injury requiring RRT, maximum 24-hour NEE dose, and maximum 24-hour lactate level. Collinearity was assessed by variance inflation factors, and model performance by the Akaike information criterion, Tjur’s coefficient of discrimination, and the proportion of correct predictions. As a post hoc stability check, backward elimination was performed from the full model; all inference is based on the prespecified full model. To identify predictors of dobutamine responsiveness, each candidate baseline clinical, hemodynamic, and echocardiographic parameter was evaluated in a separate multivariable logistic model adjusted for APACHE III score and fluid balance at the time of echocardiography. A complete case analysis was performed without imputation of missing data. All reported *p* values are two-sided, with statistical significance set at a value of less than 0.05. Analyses were performed using R (version 4.4.1; R Foundation for Statistical Computing; Vienna, Austria).

## RESULTS

In a population of 3407 patients with septic shock admitted to the ICU over the study period, a total of 251 (7.4%) received dobutamine. After application of predefined inclusion and exclusion criteria, 104 patients were included in the final analysis and 147 were excluded (**Fig. 1**). Compared with excluded dobutamine recipients, included patients more frequently had preexisting HF (60% vs. 18%; *p* < 0.001), higher peak lactate (5.7 vs. 3.1 mmol/L; *p* < 0.001), higher peak troponin T (0.17 vs. 0.06 ng/mL; *p* < 0.001), higher maximum NEE dose within 24 hours (0.69 vs. 0.28 µg/kg/min; *p* < 0.001), and a longer total duration of dobutamine infusion (1410 vs. 1025 min; *p* = 0.006). Twenty-eight-day ICU mortality (52% vs. 47%; *p* = 0.5) and hospital mortality (55% vs. 58%; *p* = 0.8) did not differ between included and excluded patients (eTable S1, https://links.lww.com/CCX/B687).

**Figure 1.**
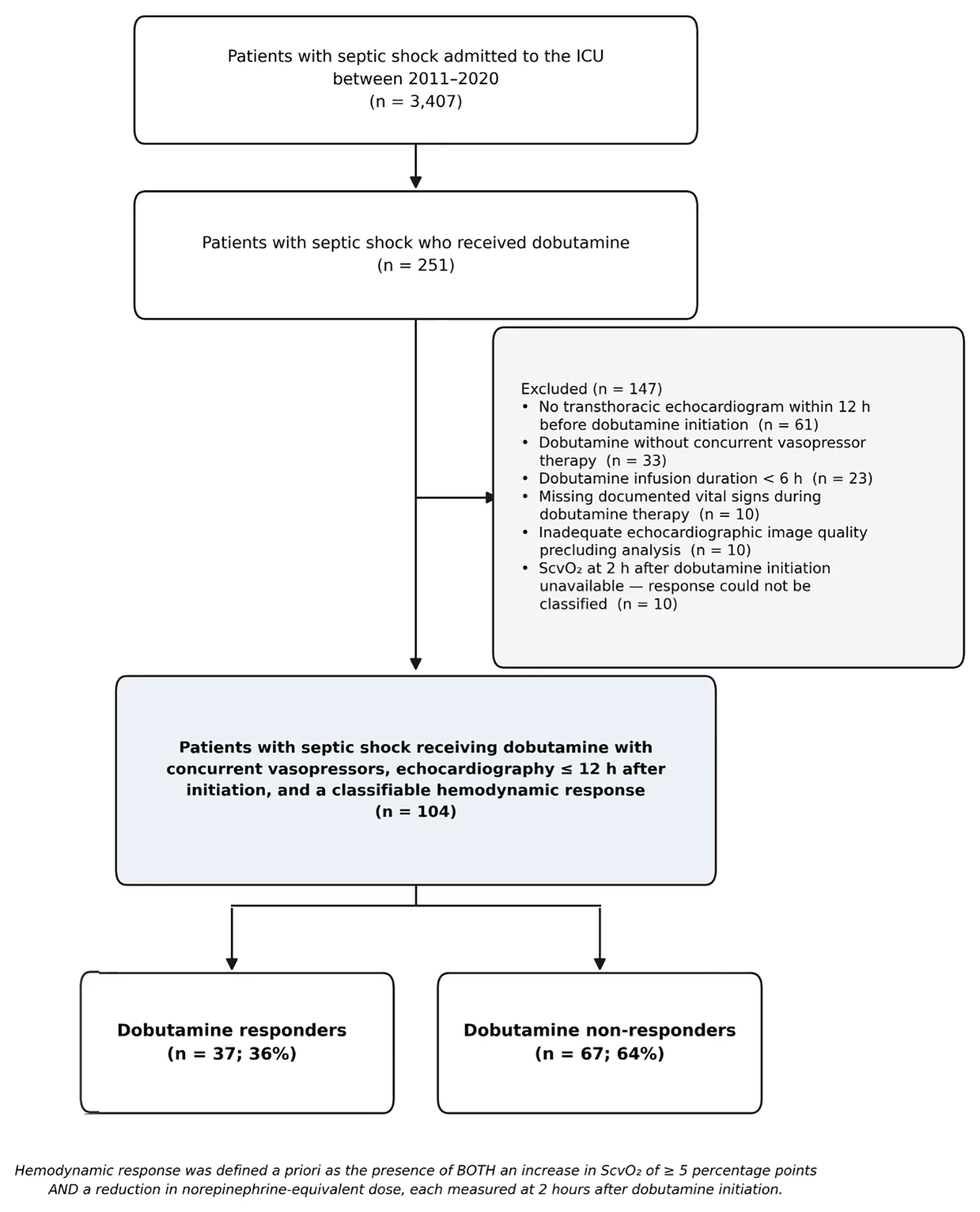
Patient selection flowchart. Identification and screening of septic shock patients admitted between 2011 and 2020, yielding 104 eligible patients receiving dobutamine, of whom 37 (36%) were classified as hemodynamic responders. ScvO_2_ = central venous oxygen saturation.

Thirty-seven (36%) were classified as dobutamine responders, whereas 67 (64%) were nonresponders. Baseline demographics and clinical characteristics are outlined in (**eTable S2**, https://links.lww.com/CCX/B687). Responders and nonresponders were similar with respect to age and sex distribution. Measures of illness severity were comparable between groups, including APACHE III score and peak lactate within the first 24 hours. The prevalence of preexisting HF did not differ significantly between responders and nonresponders, nor did the prevalence of bacteremia. The median time from echocardiography to dobutamine initiation was 3.7 hours (IQR, 1.3–10.5 hr), and the median time from ICU admission to dobutamine initiation was 21.8 hours (IQR, 2.7–80.1 hr).

Dobutamine exposure was similar between groups, including the maximum dose and the total duration of infusion (**Table 1**). At dobutamine initiation, NEE dose and the MAP/NEE ratio were similar between responders and nonresponders, whereas ScvO_2_ was lower in responders (50% vs. 54%; *p* = 0.024); lactate did not differ significantly (3.1 vs. 5.5 mmol/L; *p* = 0.058) (Table 1). By design, the groups differed at 2 hours in both components of the response definition (ΔNEE, −0.11 vs. 0.06 µg/kg/min; ΔScvO_2_, +14% vs. +4%; Table 1). Beyond the window used for classification, the separation persisted at 6 hours, with responders exhibiting a greater decrease in NEE (−0.13 [−0.22 to −0.04] vs. 0.04 [−0.02 to 0.25] µg/kg/min; *p* < 0.001), a higher MAP/NEE ratio (380 [279–899] vs. 166 [63–387]; *p* = 0.003), and a greater increase in ScvO_2_ (ΔScvO_2_, +12% vs. +6%; *p* = 0.014). In contrast, lactate did not distinguish the groups: neither the absolute change at 6 hours (−1.2 vs. −0.7 mmol/L; *p* = 0.3) nor relative lactate clearance at 6 hours (−22% vs. −15%; *p* = 0.12).

**TABLE 1.** Dobutamine Exposure, Hemodynamic and Perfusion Response, and Clinical Outcomes Among Dobutamine Responders and Nonresponders

Characteristic	Responders (*n* = 37)	Nonresponders (*n* = 67)	*p*
Dobutamine exposure			
Maximum dobutamine dose, µg/kg/min	5.0 (2.5 to 7.5)	5.0 (4.0 to 10.0)	0.2
Total dobutamine duration, min	1320 (570 to 3008)	1595 (556 to 4740)	0.5
Vasopressor requirement			
NEE at T0, µg/kg/min	0.21 (0.11 to 0.63)	0.24 (0.11 to 0.58)	> 0.9
NEE at T2, µg/kg/min^a^	0.12 (0.00 to 0.25)	0.35 (0.15 to 0.73)	0.002
NEE at T6, µg/kg/min	0.08 (0.00 to 0.22)	0.37 (0.13 to 0.80)	< 0.001
Δ NEE at T2, µg/kg/min^b^	−0.11 (−0.17 to −0.04)	0.06 (0.00 to 0.17)	—
Δ NEE at T6, µg/kg/min	−0.13 (−0.22 to −0.04)	0.04 (−0.02 to 0.25)	< 0.001
MAP/NEE at T0	364 (134 to 628)	312 (107 to 641)	0.7
MAP/NEE at T2^a^	366 (146 to 1428)	158 (85 to 403)	0.020
MAP/NEE at T6	380 (279 to 899)	166 (63 to 387)	0.003
Perfusion markers			
ScvO_2_ at T0, %	50 (38 to 55)	54 (49 to 61)	0.024
Δ ScvO_2_ at T2, %^b^	14 (10 to 17)	4 (0 to 12)	—
Δ ScvO_2_ at T6, %	12 (8 to 21)	6 (1 to 10)	0.014
Lactate at T0, mmol/L	3.1 (2.2 to 5.9)	5.5 (3.4 to 8.0)	0.058
Δ Lactate at T2, mmol/L	−0.70 (−2.05 to 0.25)	−0.30 (−1.00 to 0.70)	0.14
Δ Lactate at T6, mmol/L	−1.2 (−2.6 to −0.2)	−0.7 (−2.1 to 0.4)	0.3
Lactate clearance at T2, %	−23 (−38 to 10)	−6 (−23 to 8)	0.10
Lactate clearance at T6, %	−22 (−44 to −10)	−15 (−39 to 10)	0.12
Clinical outcomes			
ICU-free days at day 28, d^c^	17 (0 to 22)	0 (0 to 15)	0.017
AKI requiring renal replacement therapy, *n* (%)	16 (43%)	25 (37%)	0.7
28-d ICU mortality, *n* (%)	15 (41%)	39 (58%)	0.13
Hospital mortality, *n* (%)	16 (43%)	41 (61%)	0.12

AKI = acute kidney injury, MAP = mean arterial pressure, NEE = norepinephrine-equivalent, ScvO_2_ = central venous oxygen saturation, T0/T2/T6 = 0-, 2-, and 6-hr after dobutamine initiation.

a Because vasopressor dose at T0 did not differ between groups, the absolute NEE and MAP/NEE values at T2 are largely determined by the change score that forms part of the response definition, and the *p* values in these two rows should be read as partly definitional rather than as independent corroboration.

b These two variables constitute the definition of hemodynamic response (Δ ScvO_2_ ≥ 5 percentage points and Δ NEE < 0, both at 2 hr). They are shown for completeness; the between-group difference is definitional, and no *p* value is reported.

c ICU-free days at day 28 were calculated as 28 minus the number of days spent in the ICU, with patients who died before day 28 assigned zero ICU-free days.

Data are median (Q1–Q3) or *n* (%). Comparisons by Wilcoxon rank-sum test or Pearson χ^2^ test. Δ denotes change from T0.

Responders experienced more ICU-free days by day 28 (17 [0–22] vs. 0 [0–15]; *p* = 0.017). Twenty-eight-day ICU mortality was numerically lower among responders, but the difference did not reach statistical significance (15 [41%] vs. 39 [58%]; *p* = 0.13), as was the case for hospital mortality (16 [43%] vs. 41 [61%]; *p* = 0.12), and the requirement for RRT (16 [43%] vs. 25 [37%]; *p* = 0.7; Table 1). In a multivariable logistic regression model adjusting for APACHE III score, mechanical ventilation, RRT, maximum NEE dose within 24 hours, and maximum lactate within 24 hours, hemodynamic response remained independently associated with lower odds of 28-day ICU mortality (odds ratio [OR], 0.18; 95% CI, 0.04–0.68; *p* = 0.018; **Table 2**; **Fig. 2**). Variance inflation factors for all model terms were below two (**eTable S3**, https://links.lww.com/CCX/B687), and backward elimination from the full model yielded an essentially unchanged estimate (OR, 0.19; 95% CI, 0.05–0.68; *p* = 0.015; **eTable S4**, https://links.lww.com/CCX/B687).

**TABLE 2.** Multivariable Logistic Regression for 28-Day ICU Mortality

Characteristic	OR (95% CI)	*p*
Hemodynamic response (vs. nonresponder)	0.18 (0.04–0.68)	0.018
Acute Physiology and Chronic Health Evaluation III score	1.05 (1.02–1.09)	0.003
Mechanical ventilation	0.69 (0.17–2.77)	0.6
Acute kidney injury requiring renal replacement therapy	1.57 (0.46–5.77)	0.5
Maximum norepinephrine-equivalent within 24 hr, µg/kg/min	3.34 (1.53–10.10)	0.012
Maximum lactate within 24 hr, mmol/L	1.03 (0.90–1.18)	0.7

OR = odds ratio.

**Figure 2.**
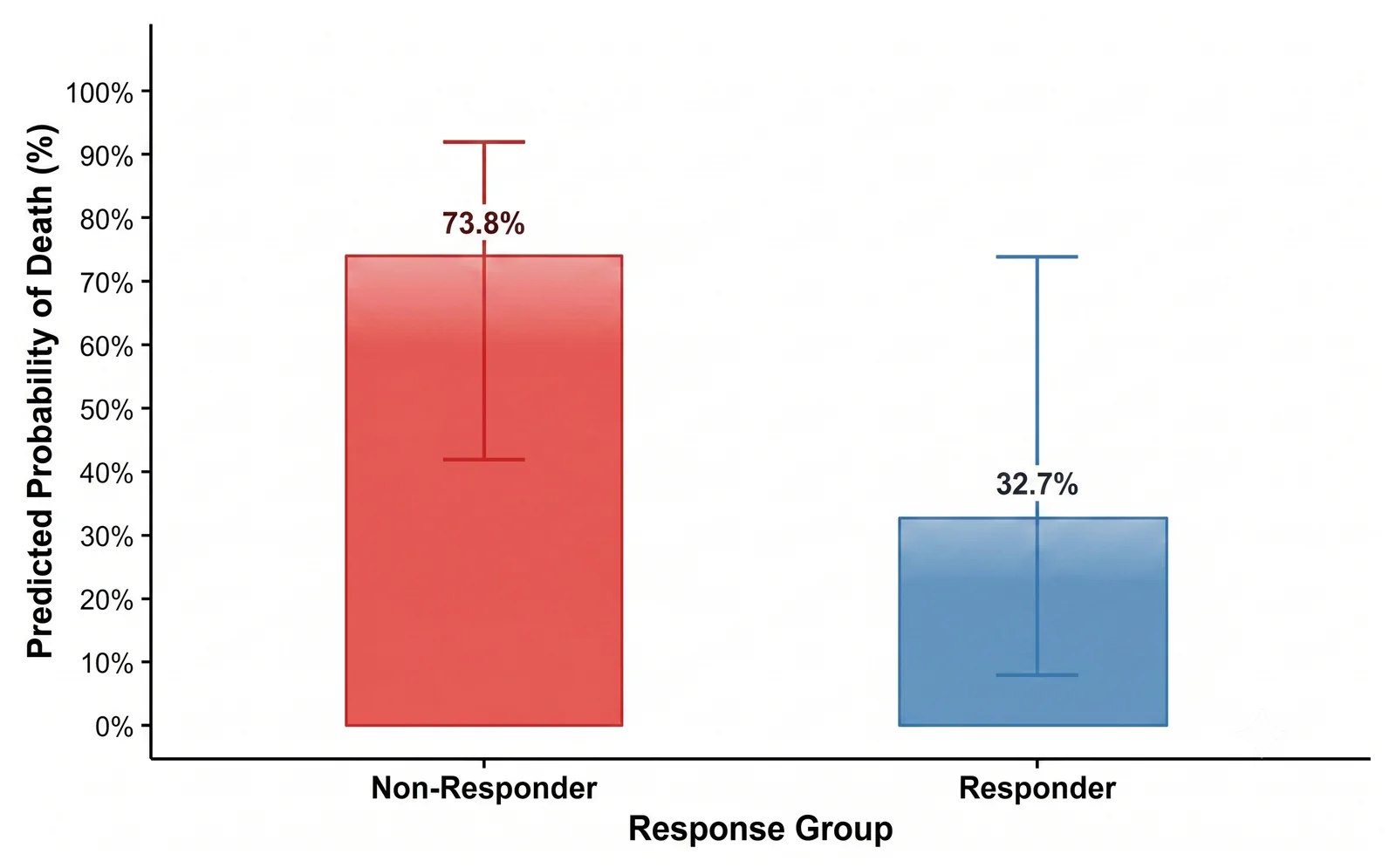
Predicted 28-day ICU mortality by Hemodynamic Response group with dobutamine. *Bar plot* illustrates adjusted probabilities of 28-d ICU mortality for nonresponders (73.8%) and responders (32.7%) following dobutamine initiation. Probabilities were derived from a multivariable logistic regression model adjusting for baseline Acute Physiology and Chronic Health Evaluation III score, mechanical ventilation, acute kidney injury requiring renal replacement therapy, maximum 24-hr norepinephrine equivalent dose, and maximum 24-hr lactate. *Bars* represent adjusted point estimates, and *error bars* indicate 95% CIs.

Responders and nonresponders demonstrated similar baseline echocardiographic and hemodynamic profiles at the time of echocardiographic assessment (**Table 3**). Heart rate, SBP, and MAP were comparable between groups, as was net fluid balance. Baseline LV systolic function was similarly impaired in both groups, with comparable LV ejection fraction, LVOT VTI, and CO. Indices of diastolic function (mitral E/A ratio, average E/e′) and of right ventricular function (tricuspid annular plane systolic excursion [TAPSE], tricuspid S′ velocity) did not differ between the groups. Afterload and ventriculo-arterial coupling measures were likewise similar before initiation of dobutamine, including SVR, Ea, Ees, and the Ees/Ea ratio.

**TABLE 3.** Echocardiographic and Hemodynamic Parameters Before Dobutamine Initiation Among Responders and Nonresponders

Characteristic	Responders (*n* = 37)	Nonresponders (*n* = 67)	*p*
Heart rate, beats/min	101 (87–121)	97 (85–114)	0.4
Systolic blood pressure, mm Hg	106 (94–114)	97 (88–112)	0.3
Mean arterial pressure, mm Hg	73 (67–81)	68 (64–76)	0.13
Fluid balance at time of echocardiography, mL	2239 (212–5107)	3537 (1122–6327)	0.3
Left ventricular ejection fraction, %	33 (25–55)	33 (25–55)	0.9
Mitral S′ lateral, m/s	0.08 (0.06–0.09)	0.07 (0.05–0.10)	0.5
Mitral S′ septal, m/s	0.06 (0.04–0.08)	0.06 (0.04–0.07)	0.7
Mitral E′ lateral, m/s	0.10 (0.08–0.12)	0.08 (0.06–0.11)	0.07
Mitral E′ septal, m/s	0.07 (0.05–0.08)	0.06 (0.04–0.08)	0.2
Mitral E/A ratio	1.38 (1.12–1.56)	1.09 (0.82–1.74)	0.4
Mitral E/E′ (average)	10 (8–12)	12 (8–16)	0.4
Left atrial volume index, mL/m^2^	29 (24–38)	26 (17–45)	0.4
Right ventricular systolic pressure, mm Hg	40 (33–49)	41 (34–50)	0.7
Tricuspid annular plane systolic excursion, cm	1.36 (1.06–1.98)	1.40 (0.99–1.87)	0.7
Tricuspid S′ lateral, cm/s	9.0 (8.0–12.9)	9.0 (6.9–11.0)	0.3
LVOT velocity–time integral, cm	11.4 (8.0–14.7)	11.7 (9.2–14.1)	0.6
LVOT stroke volume, mL	34 (23–49)	31 (25–43)	0.9
LVOT cardiac output, L/min	3.63 (2.07–5.20)	2.97 (2.46–4.29)	0.4
Systemic vascular resistance, dyn·s·cm^–5^	1310 (936–2528)	1,564 (1107–2059)	0.5
Effective arterial elastance, mm Hg/mL	2.21 (1.89–4.99)	2.80 (2.22–3.41)	0.8
End-systolic elastance, mm Hg/mL	1.74 (1.00–2.41)	1.23 (0.79–2.41)	0.7
Ventriculo-arterial coupling ratio	0.43 (0.26–1.20)	0.55 (0.25–1.55)	> 0.9

LVOT = left ventricular outflow tract.

Data are median (Q1–Q3). Comparisons by Wilcoxon rank-sum test. All measurements were obtained from the qualifying echocardiogram performed within 12 hr before dobutamine initiation.

Each baseline clinical, hemodynamic, and echocardiographic parameter was evaluated in a separate multivariable logistic regression model adjusted for APACHE III score and fluid balance at the time of echocardiography (**Table 4**). No echocardiographic parameter, including left ventricular ejection fraction (LVEF), LVOT VTI, TAPSE, tricuspid lateral S′, and effective Ea, was associated with dobutamine responsiveness. Lower ScvO_2_ at dobutamine initiation was associated with subsequent response (OR, 0.95; 95% CI, 0.91–0.99; *p* = 0.033); because baseline ScvO_2_ contributes mathematically to the change score used to define response, this association is subject to mathematical coupling and is reported as exploratory rather than as an independent prediction. Correlations among the physiologically related echocardiographic measures are shown in **eFigure S1** (https://links.lww.com/CCX/B687).

**TABLE 4.** Association Between Baseline Hemodynamic, and Echocardiographic Parameters and Hemodynamic Response to Dobutamine

Parameter	OR (95% CI)	*p*
Heart rate at T0, bpm	1.02 (0.99–1.04)	0.2
Mean arterial pressure at T0, mm Hg	1.03 (0.99–1.08)	0.2
Norepinephrine-equivalent at T0, µg/kg/min	1.22 (0.53–2.75)	0.6
Central venous oxygen saturation at T0, %	0.95 (0.91–0.99)	0.033
Lactate at T0, mmol/L	0.94 (0.83–1.05)	0.3
Left ventricular outflow tract velocity–time integral, cm	0.99 (0.90–1.08)	0.8
Left ventricular ejection fraction, %	1.00 (0.98–1.02)	> 0.9
Tricuspid annular plane systolic excursion, cm	1.10 (0.36–3.30)	0.9
Tricuspid valve lateral annular systolic velocity, cm/s	1.05 (0.93–1.19)	0.5
Effective arterial elastance, mm Hg/mL	1.07 (0.75–1.52)	0.7

OR = odds ratio, T0 = time of dobutamine initiation.

Each parameter was evaluated in a separate multivariable logistic regression model adjusted for Acute Physiology and Chronic Health Evaluation III score and fluid balance at the time of echocardiography (three terms per model).

## DISCUSSION

This retrospective study evaluated dobutamine responsiveness patterns in septic shock patients receiving echocardiography within 12 hours before initiating inotropes. Approximately one-third of patients achieved dobutamine responsiveness under our pragmatic, real-world definition: a simultaneous 5% increase in ScvO_2_ and reduced vasopressor requirements at 2 hours. Responders had more ICU-free days, and hemodynamic response was independently associated with lower adjusted odds of 28-day ICU mortality. Notably, no baseline hemodynamic or echocardiographic variable at dobutamine initiation was predictive of subsequent responsiveness.

Studies evaluating dobutamine use in septic shock have yielded heterogeneous and sometimes conflicting results (13, 20–24). Jellema et al (24) demonstrated substantial heterogeneity in hemodynamic responses to dobutamine without an associated survival benefit. Similarly, Aso et al (23) found that among adults with septic shock, dobutamine use did not affect 28-day mortality. Other observational analyses have reported an association between dobutamine use and increased mortality, raising concerns regarding potential harm (21, 22). A network meta-analysis of randomized controlled trials suggested that the addition of dobutamine to norepinephrine was associated with reduced 28-day mortality in patients with septic shock (20).

In our study, patients who demonstrated a favorable hemodynamic response to dobutamine, defined by concurrent improvement in vasopressor requirement and central venous oxygen saturation, had more favorable outcomes. Consistent with this finding, Vallet et al (25) reported that dobutamine responsiveness, defined as an increase in oxygen uptake of more than 15% during infusion, was present in 46% of patients with sepsis and was associated with improved survival. Similarly, Kumar et al (26) demonstrated that dobutamine responsiveness, defined as an increase in SV index greater than 8.5 mL/m^2^, was strongly associated with survival. Razazi et al (13) further showed in septic shock and myocardial dysfunction that dobutamine improved multiple indices of biventricular systolic and diastolic function and was associated with better outcomes among patients who tolerated therapy, including greater vasopressor-free days and lower short-term mortality. Importantly, despite differing definitions of dobutamine responsiveness across studies, ranging from the concurrent reduction in vasopressor requirement and rise in central venous oxygen saturation used in our cohort, to increased oxygen uptake, dynamic SV augmentation, and tolerance without adverse events, each identified a subset of patients in whom dobutamine use was associated with improved survival.

The heterogeneous hemodynamic responses to dobutamine in this setting are believed to be multifactorial, reflecting molecular alterations (27), varying degrees of sepsis-induced microcirculatory dysfunction, and an unpredictable interplay between β_1_-adrenergic inotropic effects and β_2_-mediated vasodilation that may exacerbate hypotension (10). Importantly, favorable responsiveness to dobutamine may reflect preserved myocardial contractile reserve and intact cellular signaling, supporting the concept that the ability to augment cardiovascular performance in response to catecholamines represents a broader physiologic reserve at the cellular level rather than isolated cardiac function (28).

Little is known about predictors of dobutamine response. Mounting evidence suggests that a low cardiac index alone does not mandate the use of dobutamine (29). Although echocardiography is essential for initial assessment, it has not been demonstrated to predict responsiveness to dobutamine (26, 29). In our study, both responders and nonresponders exhibited similarly depressed baseline LV systolic function, underscoring that resting ventricular performance does not necessarily reflect contractile reserve. Lower ScvO_2_ at the time of dobutamine initiation was associated with subsequent response, which is physiologically intuitive in that patients with the greatest baseline deficit in oxygen delivery have the most scope for improvement; we note, however, that baseline ScvO_2_ contributes to the change score by which response was defined, so this association cannot be interpreted as independent prediction. Similarly, measures of right ventricular systolic function (TAPSE, S′ wave) and ventriculo-arterial coupling (Ea/Ees) did not differ between groups. Furthermore, no single echocardiographic parameter discriminated responders from nonresponders, although with 37 responder events we cannot exclude a modest predictive association that this study was underpowered to detect. These findings may suggest that response to dobutamine represents a heterogeneous process that is poorly captured by single-point echocardiographic measurements. This is further supported by Kumar et al (26), in whom the change in SV index during a graded dobutamine challenge was the strongest hemodynamic correlate of survival; this emphasizes that myocardial contractile reserve, rather than resting function, carries prognostic information.

Nonetheless, the association between early hemodynamic response and more ICU-free days, together with the independent association with lower adjusted odds of 28-day ICU mortality, is clinically relevant as a prognostic signal. We emphasize that these findings do not establish that dobutamine improved outcome, as the observational study design in which the exposure is defined by posttreatment change cannot separate a drug effect from a more favorable underlying shock trajectory. Serial bedside reassessment incorporating echocardiography and CO monitoring may be necessary to identify patients most likely to benefit from inotropic therapy (26, 29).

This study has several limitations. First, its retrospective design introduces potential confounding; although we adjusted for multiple clinical and hemodynamic variables, unmeasured factors may have influenced outcomes, and we cannot exclude the possibility that hemodynamic responsiveness reflects greater underlying physiologic reserve rather than a direct effect of dobutamine. Because responder status was defined by changes measured after treatment began, reverse causation cannot be excluded. Several covariates in the mortality model, including mechanical ventilation, RRT, and maximum NEE dose within 24 hours, are also measured after dobutamine initiation; they were included because they are established determinants of mortality in septic shock. The findings should therefore be read as identifying a prognostic phenotype rather than as demonstrating a treatment effect. Second, echocardiography was performed on clinical indication at the treating physician’s discretion rather than as a systematic protocol, and approximately 61.8% of septic shock patients admitted during the study period underwent echocardiographic assessment. This limits generalizability to the broader septic shock population receiving inotropic therapy. Consistent with this, included patients differed from excluded dobutamine recipients in cardiac comorbidity and shock severity, although mortality was similar (eTable S1, https://links.lww.com/CCX/B687) (30, 31). Similarly, although no formal echocardiographic threshold was required for dobutamine initiation, the baseline hemodynamic and echocardiographic profiles, including depressed LVEF, reduced LVOT VTI, elevated lactate, and impaired ScvO_2_, are consistent with significant myocardial dysfunction and low CO state, supporting the clinical appropriateness of inotropic initiation in this cohort. Of note, although approximately 10% of echocardiograms performed at our institution are independently reviewed by a second blinded board-certified cardiologist, formal assessment of interobserver variability was not systematically performed. Third, standard echocardiographic parameters such as LVEF and LVOT VTI reflect resting cardiac function and may not capture myocardial contractile reserve; serial or more advanced assessments such as speckle-tracking strain echocardiography may better characterize the dynamic physiologic reserve relevant to inotrope responsiveness and warrant evaluation in future studies (32). Fourth, although our response definition pairs an objective threshold for central venous oxygen saturation improvement with vasopressor dose reduction, vasopressor titration remains subject to clinician discretion. To minimize practice variation and individual practice pattern shifts, we restricted the evaluation window to 2 hours postinitiation, increasing the likelihood that care was managed by the same clinical team. In addition, we incorporated the MAP/NEE to help account for hemodynamic fluctuations and mitigate this limitation. Fifth, because response was defined by posttreatment change, we interpret it as early hemodynamic response to dobutamine identifying a subgroup with a more favorable shock trajectory rather than establishing a treatment effect of dobutamine. Venous-to-arterial CO_2_ difference (ΔPCO_2_) may be a superior marker of improvement in cardiac function but could not be calculated in our cohort as simultaneous arterial and venous blood gas sampling was not routinely performed at our institution. In addition, continuous cardiac index monitoring was not systematically available, limiting a more comprehensive characterization of perfusion. Admittedly, continuous accounting of concurrent ICU therapies (such as hourly vasopressor titrations, fluid balance, sedation depth, and steroid administration) was unavailable in direct alignment with dobutamine dosing. While these bedside factors can theoretically influence vasopressor weaning, capturing ultra-high-frequency data across a longitudinal cohort was unachievable despite rigorous chart curation. Consequently, despite robust multivariable adjustment, residual unmeasured confounding cannot be fully excluded, representing an intrinsic constraint of observational research. We restricted inclusion to patients receiving dobutamine for at least 6 hours, which excluded those who discontinued therapy early due to intolerance or clinical deterioration; notably, no deaths occurred during this initial 6-hour period. Furthermore, detailed chart review revealed that the maximum time to early therapy discontinuation was 90 minutes. Consequently, using our 2-hour response definition, no patients were lost or excluded, ensuring complete capture of early clinical trajectories. Seventh, with 104 patients, 37 responders, and 54 deaths, statistical power was limited. Finally, this was a single-center study at a quaternary care institution, and findings may not be generalizable to other clinical settings.

## CONCLUSIONS

Early hemodynamic response to dobutamine identifies a distinct prognostic phenotype in septic shock patients with myocardial dysfunction, portending more ICU-free days and reduced adjusted 28-day mortality. Our study results underscore the prognostic value of early physiologic responsiveness. Baseline echocardiographic parameters lacked predictive value for dobutamine responsiveness. Whether serial or dynamic hemodynamic assessment can better stratify patients likely to benefit from dobutamine warrants prospective investigation.

## Supplementary Material
