# Supplementary material for: Response to Dobutamine in Septic Shock With Myocardial Dysfunction: A Single-Center Retrospective Study
Source: Crit Care Explor. 2026 Sep 25;8(10):e1487. doi: 10.1097/CCE.0000000000001487 (PMC13619267; doi:10.1097/CCE.0000000000001487)
Supplement: Supplementary file 1 [file cc9-8-e1487-s001.pdf]

Supplemental Digital Content

Response to Dobutamine in Septic Shock with Myocardial Dysfunction: A Single-Center Retrospective Study

Table of Contents

eTable S1. Comparison of dobutamine recipients included in and excluded from the analytic cohort.

eTable S2. Baseline demographics and clinical characteristics of dobutamine responders and non-responders.

eTable S3. Model diagnostics: variance inflation factors and model performance.

eTable S4. Reduced model after backward elimination (post hoc stability check).

eFigure S1. Correlation matrix of clinical, hemodynamic, and echocardiographic variables.

Item 1. STROBE Statement checklist.

eTable S1. Comparison of dobutamine recipients included in and excluded from the analytic cohort.

Abbreviations: AKI: acute kidney injury; APACHE III: Acute Physiology and Chronic Health Evaluation III; BMI: body mass index; BUN: blood urea nitrogen; COPD: chronic obstructive pulmonary disease; ICU: intensive care unit; NEE: norepinephrine-equivalent; RRT: renal replacement therapy; WBC: white blood cell count.

Characteristic	All recipients (n = 251)	Excluded (n = 147)	Included (n = 104)	p-value
**OUTCOMES**				
28-day ICU mortality, n (%)	123 (49%)	69 (47%)	54 (52%)	0.5
Hospital mortality, n (%)	143 (57%)	86 (59%)	57 (55%)	0.8
**DEMOGRAPHICS**				
Age, years	66 (55, 74)	65 (57, 74)	67 (52, 76)	0.8
Female sex, n (%)	125 (50%)	73 (50%)	52 (50%)	>0.9
BMI, kg/m^2	28 (25, 33)	28 (24, 34)	28 (25, 31)	0.3
Height, cm	168 (163, 178)	170 (163, 178)	168 (160, 178)	0.6
Weight, kg	79 (68, 97)	79 (70, 99)	79 (67, 96)	0.4
**SOURCE OF INFECTION**				0.4
Pulmonary	87 (35%)	44 (30%)	43 (41%)	
Abdominal	48 (19%)	29 (20%)	19 (18%)	
Urological	29 (12%)	18 (12%)	11 (11%)	
Skin/soft tissue	20 (8%)	14 (10%)	7 (7%)	
Line-related	12 (5%)	10 (7%)	2 (2%)	
Other	54 (22%)	32 (22%)	22 (21%)	
**SEVERITY AND COMORBIDITIES**				
APACHE III score	105 (83, 132)	105 (78, 137)	106 (89, 129)	0.3
Heart failure, n (%)	88 (35%)	26 (18%)	62 (60%)	<0.001
Cirrhosis, n (%)	22 (9%)	18 (12%)	4 (4%)	0.061
COPD, n (%)	64 (26%)	40 (27%)	24 (23%)	0.6
Diabetes, n (%)	89 (35%)	50 (34%)	39 (38%)	0.7
Chronic dialysis, n (%)	41 (16%)	25 (17%)	16 (15%)	0.8
Immune suppression, n (%)	61 (24%)	35 (24%)	26 (25%)	0.9
Malignancy, n (%)	42 (17%)	22 (15%)	20 (19%)	0.5
**LABORATORY VALUES, VITALS, AND THERAPY**				

Characteristic	All recipients (n = 251)	Excluded (n = 147)	Included (n = 104)	p-value
Urine output (24 h), mL	472 (107, 1,183)	349 (35, 995)	723 (258, 1,401)	0.002
BUN, mg/dL	42 (27, 61)	39 (25, 59)	44 (28, 68)	0.13
Creatinine, mg/dL	2.18 (1.35, 3.39)	2.14 (1.42, 3.19)	2.26 (1.28, 3.71)	0.3
Albumin, g/dL	2.30 (2.00, 2.80)	2.40 (1.90, 2.80)	2.30 (2.10, 2.70)	0.7
Bilirubin, mg/dL	1.1 (0.5, 2.3)	1.1 (0.5, 2.6)	1.0 (0.5, 2.1)	0.5
Peak lactate (24 h), mmol/L	4.2 (1.9, 8.4)	3.1 (1.3, 6.4)	5.7 (2.7, 10.3)	<0.001
Peak troponin T (7 d), ng/mL	0.11 (0.03, 0.33)	0.06 (0.01, 0.27)	0.17 (0.07, 0.36)	<0.001
Maximum NEE (24 h), mcg/kg/min	0.54 (0.17, 1.02)	0.28 (0.08, 0.84)	0.69 (0.34, 1.26)	<0.001
Fluid balance at echocardiography, mL	2,707 (883, 5,718)	2,290 (762, 3,967)	2,903 (1,122, 5,956)	0.3
Mechanical ventilation, n (%)	177 (71%)	99 (67%)	78 (75%)	0.2
AKI requiring RRT, n (%)	111 (44%)	69 (47%)	42 (40%)	0.4
Maximum dobutamine dose (24 h), mcg/kg/min	5.0 (2.5, 7.5)	5.0 (2.5, 6.0)	5.0 (3.0, 8.0)	0.034
Dobutamine duration, min	1,260 (252, 3,459)	1,025 (80, 3,414)	1,410 (551, 3,645)	0.006
Hospital-free days	0 (0, 4)	0 (0, 4)	0 (0, 10)	0.4
ICU-free days	0 (0, 17)	0 (0, 13)	0 (0, 18)	0.2

Data are median (Q1, Q3) or n (%). p-values compare included with excluded recipients by Wilcoxon rank-sum test, Pearson chi-squared test, or Fisher exact test as appropriate. Echocardiographic parameters are not tabulated for excluded patients because the largest single exclusion category (n = 61) was the absence of a qualifying echocardiogram; fluid balance at the time of echocardiography is reported for the subset of excluded patients in whom a non-qualifying echocardiogram was nevertheless performed. Ten patients who met all other eligibility criteria but in whom central venous oxygen saturation at 2 hours was unavailable could not be classified as responders or non-responders and are counted among the excluded.

eTable S2. Baseline demographics and clinical characteristics of dobutamine responders and non-responders.

Characteristic	Responders (n = 37)	Non-Responders (n = 67)	p-value
**Age, years**	69 (58, 74)	66 (50, 76)	0.7
**Female sex, n (%)**	17 (46%)	35 (52%)	0.5
**BMI, kg/m^2**	28 (23, 31)	28 (26, 31)	>0.9
**APACHE III score**	111 (92, 129)	101 (89, 129)	0.6
**Heart failure, n (%)**	19 (51%)	37 (55%)	0.7
**Cirrhosis, n (%)**	1 (2.7%)	4 (6.0%)	0.7
**COPD, n (%)**	10 (27%)	17 (25%)	0.9
**Diabetes mellitus, n (%)**	14 (38%)	23 (34%)	0.7
**Chronic dialysis, n (%)**	5 (14%)	12 (18%)	0.6
**Albumin, g/dL**	2.30 (2.00, 2.70)	2.30 (2.10, 2.70)	0.8
**Bilirubin, mg/dL**	1.00 (0.60, 2.10)	1.10 (0.50, 2.00)	0.8
**Peak serum lactate within 24 h, mmol/L**	3.9 (2.7, 8.4)	5.7 (2.5, 10.8)	0.2
**Bacteremia, n (%)** [a]	11 (34%)	18 (31%)	0.7
**Site of infection, n (%)** [a]			0.3
Pulmonary	11 (34%)	20 (34%)	
Urological	5 (16%)	5 (8.5%)	
Skin/soft tissue	1 (3.1%)	4 (6.8%)	
Abdominal	7 (22%)	5 (8.5%)	
Line-related	0 (0%)	2 (3.4%)	
Other	8 (25%)	23 (39%)	

Data are median (Q1, Q3) or n (%). Comparisons by Wilcoxon rank-sum test, Pearson chi-squared test, or Fisher exact test as appropriate. No values were imputed.

[a] Denominators differ from the group totals because these variables were incompletely documented. Site of infection was recorded for 32 of 37 responders and 59 of 67 non-responders; bacteremia was recorded for 32 of 37 responders and 58 of 67 non-responders. Percentages are calculated on the documented denominators.

Abbreviations: APACHE III: Acute Physiology and Chronic Health Evaluation III; BMI: body mass index; COPD: chronic obstructive pulmonary disease; ICU: intensive care unit; IQR: interquartile range

eTable S3. Model diagnostics: variance inflation factors and model performance.

Abbreviations: AKI: acute kidney injury; APACHE III: Acute Physiology and Chronic Health Evaluation III; CI: confidence interval; ICU: intensive care unit; NEE: norepinephrine-equivalent; OR: odds ratio; RRT: renal replacement therapy.

Panel A. Variance inflation factors

Model term	Variance inflation factor
**Hemodynamic response**	1.29
**APACHE III score**	1.24
**Mechanical ventilation**	1.11
**AKI requiring RRT**	1.14
**Maximum NEE within 24 h**	1.12
**Maximum lactate within 24 h**	1.09

All variance inflation factors were below 2, indicating no material collinearity among model terms.

Panel B. Model performance

Metric	Value
**Akaike information criterion (AIC)**	84.3
**Corrected AIC (AICc)**	85.9
**Bayesian information criterion (BIC)**	100.9
**Tjur's coefficient of discrimination (R^2)**	0.407
**Root mean square error**	0.386
**Logarithmic loss**	0.445
**Proportion of correct predictions**	0.704

eTable S4. Reduced model for 28-day ICU mortality after backward elimination.

Characteristic	OR	95% CI	p-value
**Hemodynamic response (vs non-responder)**	0.19	0.05, 0.68	0.015
**APACHE III score, per 1 point**	1.05	1.02, 1.09	0.003
**Maximum NEE within 24 h, per 1 mcg/kg/min**	3.20	1.54, 9.06	0.010

Backward elimination by Akaike information criterion, beginning from the full model in Table 2. The estimate for hemodynamic response was essentially unchanged (OR 0.18 to 0.19), indicating that the association does not depend on the particular covariate set. This reduced model is presented as a post hoc stability check only; all inference is based on the prespecified full model in Table 2.

eFigure S1. Correlation matrix of clinical, hemodynamic, and echocardiographic variables.

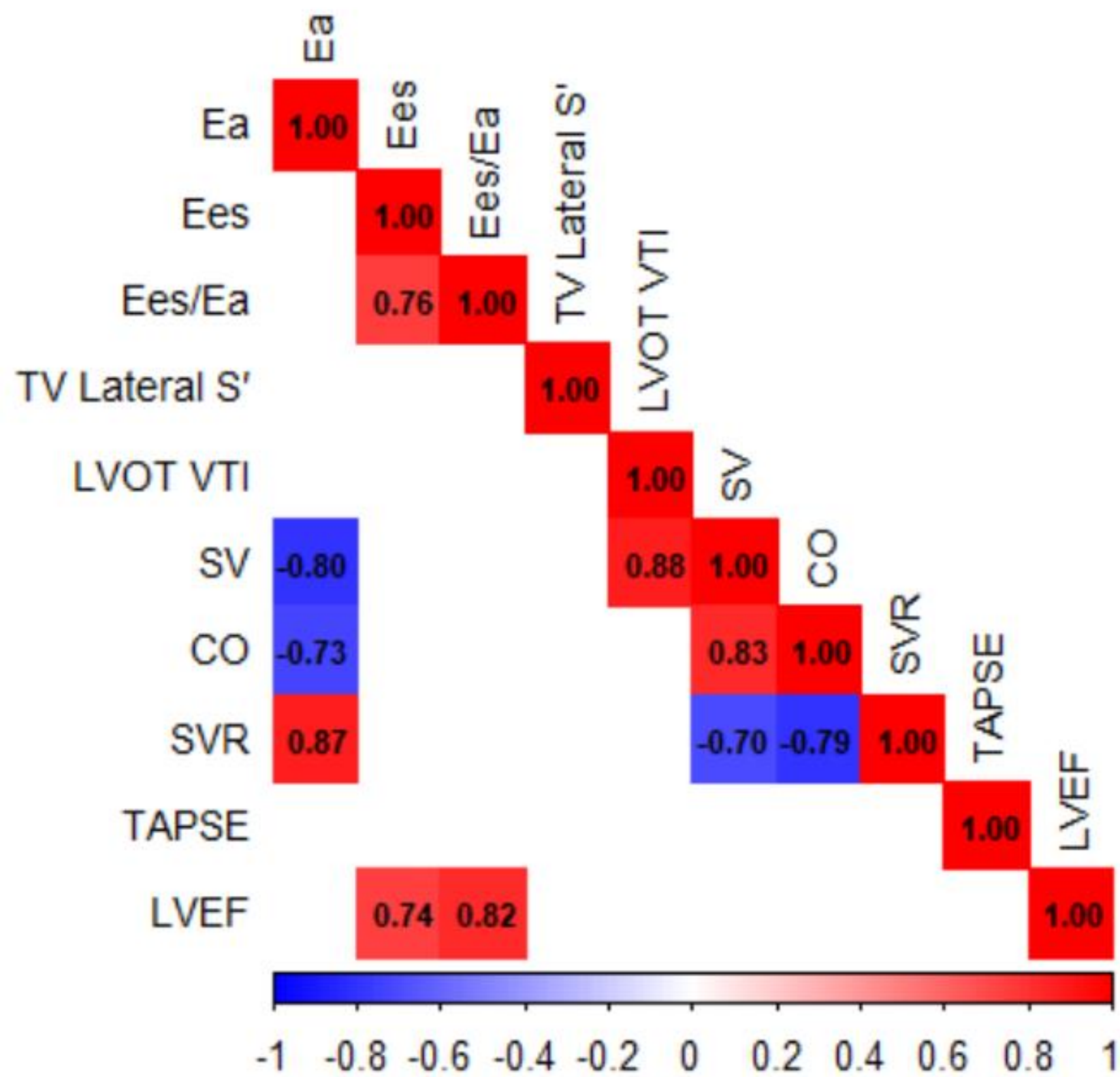

Abbreviations: CO: cardiac output; Ea: effective arterial elastance; Ees: left ventricular end-systolic elastance; Ees/Ea: ventriculo-arterial coupling ratio; LVEF: left ventricular ejection fraction; LVOT VTI: left ventricular outflow tract velocity–time integral; SV: stroke volume; SVR: systemic vascular resistance; TAPSE: tricuspid annular plane systolic excursion; TV lateral S′: tricuspid valve lateral annular systolic velocity.

Item 1. STROBE Statement Checklist

STROBE Statement — checklist of items that should be included in reports of observational studies (cohort studies)

	Item No	Recommendation and location in the revised manuscript	Page No
Title and abstract			
**Title and abstract**	**1**	*(a)* Indicate the study's design with a commonly used term in the title or the abstract. **Reported as "Single-Center Retrospective Study" in the title, and as "Single-center retrospective cohort study" in the abstract under Design, Setting, and Participants.**	1, 5
		(b) Provide in the abstract an informative and balanced summary of what was done and what was found. **Structured abstract (Importance; Objectives; Design, Setting, and Participants; Main Outcomes and Measures; Results; Conclusions and Relevance). The abstract reports the non-significant unadjusted mortality difference (41% vs 58%, p = 0.13) alongside the adjusted estimate, states that the design cannot establish causation, and states that the findings are hypothesis-generating. Key Points (Question, Findings, Meaning) follow the abstract.**	5–7
Introduction			
**Background/rationale**	**2**	Explain the scientific background and rationale for the investigation being reported. **Introduction: epidemiology and prognostic significance of sepsis-induced myocardial dysfunction; prognostic weight of pre-existing heart failure; the low-quality evidence base underlying the Surviving Sepsis Campaign suggestion for dobutamine; heterogeneity of hemodynamic response and blunted adrenergic responsiveness in sepsis; absence of any established phenotype**	8–9

	Item No	Recommendation and location in the revised manuscript	Page No
		**identifying likely responders.**	
**Objectives**	**3**	State specific objectives, including any prespecified hypotheses. **Stated at the end of the Introduction: “we sought to determine whether clinical outcomes differ between dobutamine responders and non-responders, and if any baseline echocardiographic and physiologic parameters might predict hemodynamic response following dobutamine initiation.” Aligned objectives appear in the abstract (Objectives) and in Key Points (Question).**	5, 7, 9
Methods			
**Study design**	**4**	Present key elements of study design early in the paper. **Single-center retrospective cohort study, identified in the abstract Design and in the opening sentence of Methods.**	5, 10
**Setting**	**5**	Describe the setting, locations, and relevant dates, including periods of recruitment, exposure, follow-up, and data collection. **Medical ICU of a quaternary academic medical center (Cleveland Clinic), 2011 through 2020. Exposure anchored at dobutamine initiation; hemodynamic and perfusion variables collected at 0, 2 and 6 hours after initiation; outcomes ascertained from the electronic medical record through 28 days from dobutamine initiation and through the index hospitalization for hospital mortality.**	10
**Participants**	**6**	*(a) Cohort study* — Give the eligibility criteria, and the sources and methods of selection of participants. Describe methods of follow-up. **Adults (≥ 18 years) admitted to the ICU with septic shock by Sepsis-3.0 criteria who received dobutamine with concurrent vasopressor therapy and underwent transthoracic echocardiography during catecholamine administration within 12 hours before**	10–11, Fig 1

	Item No	Recommendation and location in the revised manuscript	Page No
		**dobutamine initiation. Exclusions: severe valvular disease; history of heart or lung transplantation; moderate or large pericardial effusion; unavailable hourly vasopressor and inotrope data; dobutamine without concurrent vasopressor therapy; dobutamine discontinued within 6 hours of initiation; and central venous oxygen saturation at 2 hours unavailable, precluding classification. Cohort flow is shown in Figure 1. Outcomes were ascertained from the electronic medical record; there was no loss to follow-up.**	
		(b) Cohort study — For matched studies, give matching criteria and number of exposed and unexposed. **Not applicable. This is an unmatched single-arm cohort; comparisons are between dobutamine responders (n = 37) and non-responders (n = 67) within the cohort.**	N/A
**Variables**	**7**	Clearly define all outcomes, exposures, predictors, potential confounders, and effect modifiers. Give diagnostic criteria, if applicable. **Exposure: dobutamine, initiated at the treating physician's discretion with no predefined echocardiographic threshold. Classification variable: hemodynamic response, defined a priori as the presence of BOTH an increase in central venous oxygen saturation of at least 5 percentage points AND a reduction in norepinephrine-equivalent dose, each measured at 2 hours after dobutamine initiation; patients not meeting both criteria were classified as non-responders. Septic shock defined by Sepsis-3.0 criteria. Norepinephrine-equivalent dose, stroke volume, arterial elastance, end-systolic elastance, the Ees/Ea ratio, and systemic vascular resistance are defined by explicit**	10–13

	Item No	Recommendation and location in the revised manuscript	Page No
		**formulas in Methods. Primary outcome: 28-day ICU mortality from dobutamine initiation. Other outcomes: ICU-free days at day 28, acute kidney injury requiring renal replacement therapy, hospital mortality. Confounders adjusted for in the mortality model: APACHE III score, mechanical ventilation, acute kidney injury requiring renal replacement therapy, maximum norepinephrine-equivalent dose within 24 hours, maximum lactate within 24 hours. Confounders adjusted for in the models of response: APACHE III score and fluid balance at the time of echocardiography. No effect modifiers were prespecified.**	
**Data sources/measurement**	**8**	For each variable of interest, give sources of data and details of methods of assessment. Describe comparability of assessment methods if there is more than one group. **Demographics, comorbidities, illness severity scores, and hemodynamic and laboratory data were extracted from the electronic medical record. Echocardiograms were performed by registered cardiac sonographers using commercially available systems; all measurements were made offline by board-certified cardiologists using Syngo Dynamics (Siemens Healthcare) according to American Society of Echocardiography guidelines. Approximately 10% of studies at the institution undergo independent review by a second blinded board-certified cardiologist, but formal interobserver variability was not assessed. Assessment methods were identical for responders and non-responders, and group assignment was made after all measurements had been recorded.**	11–12
**Bias**	**9**	Describe any efforts to address potential sources of bias. **Selection bias: inclusion**	10–13, 22–24

	Item No	Recommendation and location in the revised manuscript	Page No
		**restricted to patients with an echocardiogram performed during catecholamine administration within 12 hours before dobutamine initiation, and characterised quantitatively by comparing all 251 dobutamine recipients, the 147 excluded, and the 104 analysed (eTable S1). Confounding: multivariable logistic regression with covariates prespecified on clinical grounds rather than by data-driven screening; collinearity assessed by variance inflation factors (eTable S3). Circularity: the two variables constituting the response definition are reported without p-values in Table 2, and evidence that the classification captures a physiological difference is drawn from the 6-hour timepoint, outside the classification window. Residual bias — confounding by indication, clinically indicated rather than protocolised echocardiography, clinician-influenced vasopressor titration, adjustment for post-treatment covariates, survivor and responder bias from the 6-hour exposure requirement, and secular change over a ten-year period — is set out in Limitations.**	
**Study size**	**10**	Explain how the study size was arrived at. **No a priori sample size calculation was performed. The study included every eligible patient with septic shock who received dobutamine at the institution between 2011 and 2020; the final analytic cohort was 104 patients with 37 responders and 54 deaths. This convenience sample is intrinsic to the retrospective single-center design, and the resulting limitation of statistical power is stated explicitly in Limitations and in the abstract Conclusions.**	15, Fig 1, 24
**Quantitative variables**	**11**	Explain how quantitative variables were handled in the analyses. If applicable,	12–13

	Item No	Recommendation and location in the revised manuscript	Page No
		describe which groupings were chosen and why. **All continuous variables were analysed on their original continuous scale in all regression models; none was categorised. The single dichotomy in the analysis is the responder classification itself, defined by the two thresholds given under item 7 ($ScvO_2$ increase ≥ 5 percentage points and any reduction in norepinephrine-equivalent dose, both at 2 hours). The dichotomisation of LVOT VTI and TAPSE that appeared in the original submission has been removed.**	
**Statistical methods**	**12**	*(a)* Describe all statistical methods, including those used to control for confounding. **Continuous variables are summarised as median (25th, 75th percentile) and compared with the Wilcoxon rank-sum (Mann–Whitney U) test; categorical variables are compared with the Pearson chi-squared or Fisher exact test as appropriate. The association between hemodynamic response and 28-day ICU mortality was estimated by multivariable logistic regression with the five prespecified covariates listed under item 7; Sequential Organ Failure Assessment score was deliberately excluded because of substantial collinearity with APACHE III. Predictors of response were assessed by fitting each candidate baseline parameter in a separate multivariable logistic model adjusted for APACHE III score and fluid balance at the time of echocardiography (three terms per model). All p-values are two-sided with significance set at 0.05. Analyses were performed in R version 4.4.1.**	12–13
		(b) Describe any methods used to examine subgroups and interactions. **No subgroup analyses and no interaction terms were tested. The LVOT VTI × TAPSE interaction reported in the original submission has**	13

	Item No	Recommendation and location in the revised manuscript	Page No
		**been withdrawn.**	
		(c) Explain how missing data were addressed. **No imputation was performed; all analyses are complete-case. Ten patients who met all other eligibility criteria but in whom central venous oxygen saturation at 2 hours was unavailable could not be classified and were excluded rather than imputed (Figure 1). The multivariable mortality model was fitted on patients with complete data for all model covariates. Each single-predictor model of response was fitted on the patients with a non-missing value for that predictor, and the number contributing to each model is reported in Table 4. The multiple-imputation procedure described in the original submission was not used in the revised analysis and has been removed from the Methods.**	13
		(d) Cohort study — If applicable, explain how loss to follow-up was addressed. **Not applicable. All outcomes were ascertained from institutional electronic medical records during the index hospitalisation, with mortality follow-up complete through 28 days from dobutamine initiation. No patient was lost to follow-up for any reported outcome.**	13
		(e) Describe any sensitivity analyses. **Backward elimination from the full prespecified mortality model was performed as a post hoc stability check; the estimate for hemodynamic response was essentially unchanged (eTable S4). Variance inflation factors and model performance indices are reported in eTable S3. Correlations among physiologically related echocardiographic measures are shown in eFigure S1.**	13
Results			
**Participants**	**13**	*(a)* Report numbers of individuals at each	15, Fig 1

	Item No	Recommendation and location in the revised manuscript	Page No
		stage of study. **3,407 patients with septic shock were admitted to the ICU during the study period; 251 (7.4%) received dobutamine; 147 were excluded and 104 were included in the analytic cohort, of whom 37 (36%) were classified as responders and 67 (64%) as non-responders. All 104 contributed to the primary outcome analysis.**	
		(b) Give reasons for non-participation at each stage. **Reasons for exclusion, with the number excluded for each, are shown in Figure 1: no transthoracic echocardiogram within 12 hours before dobutamine initiation (n = 61); dobutamine without concurrent vasopressor therapy (n = 33); dobutamine infusion duration under 6 hours (n = 23); missing documented vital signs during dobutamine therapy (n = 10); inadequate echocardiographic image quality (n = 10); central venous oxygen saturation at 2 hours unavailable (n = 10).**	Fig 1
		(c) Consider use of a flow diagram. **A cohort flow diagram is provided as Figure 1.**	Fig 1
**Descriptive data**	**14**	*(a)* Give characteristics of study participants and information on exposures and potential confounders. **Baseline demographics, comorbidities, illness severity and laboratory values, together with timing of echocardiography and of dobutamine initiation, are reported in Table 1. Dobutamine exposure, vasopressor requirement, and perfusion markers at 0, 2 and 6 hours are reported in Table 2. Baseline hemodynamic and echocardiographic profiles are reported in Table 3. The analytic cohort is compared with excluded dobutamine recipients in eTable S1.**	15–17
		(b) Indicate number of participants with missing data for each variable of interest.	15–17, eTables

	Item No	Recommendation and location in the revised manuscript	Page No
		**Tables 1–3 report complete-case statistics; where a variable was incompletely recorded, the denominator contributing to each comparison is given in the table footnote. Site of infection and bacteraemia were documented for a subset of the cohort, and the contributing denominators are footnoted in Table 1. The number of patients contributing to each single-predictor model of response is reported in the corresponding row of Table 4. The number of patients contributing to the multivariable mortality model is reported in the footnote to eTable S2 and in eTable S3 Panel B. No values were imputed.**	S2–S3
		(c) Cohort study — Summarise follow-up time. **Follow-up for the primary outcome was fixed at 28 days from dobutamine initiation for every patient. Hospital mortality was ascertained through the index hospitalisation. Median total dobutamine infusion duration was 1,320 minutes in responders and 1,595 minutes in non-responders (Table 2).**	15–16
**Outcome data**	**15**	*Cohort study* — Report numbers of outcome events or summary measures over time. **Primary outcome, 28-day ICU mortality: 15 of 37 responders (41%) versus 39 of 67 non-responders (58%), p = 0.13; 54 deaths in total. Hospital mortality: 16 of 37 (43%) versus 41 of 67 (61%), p = 0.12. Acute kidney injury requiring renal replacement therapy: 16 of 37 (43%) versus 25 of 67 (37%), p = 0.7. ICU-free days at day 28: median 17 (IQR 0–22) versus 0 (IQR 0–15), p = 0.017. All outcome data are reported in Table 2 and in the Results text.**	16, Table 2
**Main results**	**16**	*(a)* Give unadjusted estimates and, if applicable, confounder-adjusted estimates and their precision. Make clear which confounders were adjusted for and why.	16–17, eTables S2–S3

	Item No	Recommendation and location in the revised manuscript	Page No
		**Unadjusted between-group comparisons with p-values are reported in Tables 1–3. The adjusted estimate for the primary outcome is an odds ratio of 0.18 (95% CI 0.04–0.68, p = 0.018) for hemodynamic response, from a multivariable logistic model adjusting for APACHE III score, mechanical ventilation, acute kidney injury requiring renal replacement therapy, maximum norepinephrine-equivalent dose within 24 hours, and maximum lactate within 24 hours (eTable S2). These covariates were prespecified on clinical grounds as established determinants of mortality in septic shock and, in the case of mechanical ventilation and renal replacement therapy, in direct response to editorial comment; the Limitations state explicitly that three of them are ascertained after dobutamine initiation and that adjustment for post-treatment variables may bias the estimate in either direction. Collinearity was excluded by variance inflation factors, all below 2 (eTable S3). Ten baseline parameters were each evaluated in a separate adjusted model as predictors of response (Table 4), without adjustment for multiplicity, and this is stated in the Results.**	
		(b) Report category boundaries when continuous variables were categorized. **No continuous variable was categorised in the revised analysis. The two thresholds defining the responder classification are an increase in central venous oxygen saturation of at least 5 percentage points and any reduction in norepinephrine-equivalent dose, both measured at 2 hours after dobutamine initiation; the rationale for the 5-percentage-point threshold is given in Methods.**	12
		(c) If relevant, consider translating estimates	16,

	Item No	Recommendation and location in the revised manuscript	Page No
		of relative risk into absolute risk for a meaningful time period. **Absolute event rates are reported alongside the adjusted estimate: 28-day ICU mortality was 41% in responders and 58% in non-responders, an absolute difference of approximately 17 percentage points over 28 days, which did not reach statistical significance in a cohort of this size (p = 0.13).**	Table 2
**Other analyses**	**17**	Report other analyses done — eg analyses of subgroups and interactions, and sensitivity analyses. **Ten separate adjusted logistic models of baseline clinical, hemodynamic and echocardiographic parameters as predictors of hemodynamic response (Table 4). Comparison of included with excluded dobutamine recipients (eTable S1). Model diagnostics comprising variance inflation factors and performance indices (eTable S3). Backward elimination from the full model as a post hoc stability check (eTable S4). Correlation matrix of physiologically related measures (eFigure S1).**	17, eTables S1–S5
Discussion			
**Key results**	**18**	Summarise key results with reference to study objectives. **The opening paragraph of the Discussion states that 36% of patients met the composite response criteria; that responders had more ICU-free days and lower adjusted odds of 28-day ICU mortality while the unadjusted mortality difference did not reach significance and renal replacement therapy did not differ; that these differences occurred despite similar baseline demographics, illness severity, vasopressor dose and echocardiographic profiles; and that, because response was defined by post-treatment change, the finding identifies a subgroup with a more favourable shock trajectory rather than establishing a**	18

	Item No	Recommendation and location in the revised manuscript	Page No
		**treatment effect of dobutamine.**	
**Limitations**	**19**	Discuss limitations of the study, taking into account sources of potential bias or imprecision. Discuss both direction and magnitude of any potential bias. **Limitations are set out in order: (1) retrospective design with residual confounding, explicit acknowledgement that reverse causation cannot be excluded because responder status was defined after treatment began, and explicit acknowledgement that three model covariates are post-treatment and may bias the estimate in either direction; (2) selection bias from clinically indicated rather than protocolised echocardiography, quantified against excluded recipients in eTable S1, and absence of systematic interobserver assessment; (3) resting echocardiographic parameters do not capture contractile reserve; (4) the response definition relies partly on clinician-influenced vasopressor titration, both components are measured at a single timepoint, and serial lactate did not corroborate the classification at any timepoint; (5) venous-to-arterial CO_2 difference could not be calculated and continuous cardiac index monitoring was unavailable; (6) survivor and responder bias arising from the 6-hour minimum exposure requirement; (7) limited statistical power with 104 patients, 37 responders and 54 deaths; (8) secular change in sepsis definitions and resuscitation practice across the 2011–2020 period; (9) absence of co-intervention data; (10) single-centre quaternary setting.**	22–24
**Interpretation**	**20**	Give a cautious overall interpretation of results considering objectives, limitations, multiplicity of analyses, results from similar studies, and other relevant evidence. **The Discussion positions the findings against**	18–25

	Item No	Recommendation and location in the revised manuscript	Page No
		**prior work (Jellema, Aso, Vallet, Kumar, Razazi), notes that studies using different definitions of responsiveness have each identified a subgroup with better outcome, and offers mechanistic context (heterogeneous adrenergic signalling, microcirculatory dysfunction, contractile reserve). The interpretation offered is deliberately restricted: early hemodynamic response identifies a prognostic phenotype rather than demonstrating benefit from dobutamine; resting echocardiography failed to identify responders, expressed as a failure to detect a predictor rather than as evidence that none exists; the association with baseline $ScvO_2$ is flagged as subject to mathematical coupling and to multiplicity and is not presented as prediction; and the findings are stated to be hypothesis-generating and to require prospective confirmation.**	
**Generalisability**	**21**	Discuss the generalisability (external validity) of the study results. **Addressed in Limitations: a single quaternary academic centre; echocardiography performed on clinical indication rather than by protocol, covering approximately 62% of septic shock admissions during the study period; dobutamine initiated at clinician discretion; and an analytic cohort demonstrably enriched for pre-existing heart failure and more severe shock relative to excluded dobutamine recipients (eTable S1). Generalisability to the broader septic shock population receiving inotropic therapy is therefore explicitly limited.**	22–24
Other information			
**Funding**	**22**	Give the source of funding and the role of the funders for the present study and, if applicable, for the original study on which the present article is based. **“The authors**	2, 26

	Item No	Recommendation and location in the revised manuscript	Page No
		**received no financial support for the research, authorship, or publication of this article." Stated on the title page and repeated in the Funding statement. No funder had any role in the design, conduct, analysis, or reporting of the study.**	

STROBE checklist adapted from von Elm E, Altman DG, Egger M, Pocock SJ, Gøtzsche PC, Vandenbroucke JP; STROBE Initiative. The Strengthening the Reporting of Observational Studies in Epidemiology (STROBE) statement: guidelines for reporting observational studies. Lancet. 2007;370(9596):1453–1457.
